# Editorial: Perinatal hypoxic-ischemic brain injury: Mechanisms, pathogenesis, and potential therapeutic strategies

**DOI:** 10.3389/fncel.2022.1086692

**Published:** 2022-12-13

**Authors:** Xiaodi Chen, Shadi Nawaf Malaeb, Jonathan Pan, Laishuan Wang, Joseph Scafidi

**Affiliations:** ^1^Women and Infants Hospital of RI, Alpert Medical School of Brown University, Providence, RI, United States; ^2^College of Medicine, Drexel University, Philadelphia, PA, United States; ^3^Department of Anesthesia and Perioperative Care, University of California, San Francisco, San Francisco, CA, United States; ^4^Children's Hospital, Fudan University, Shanghai, China; ^5^Department of Neurology and Pediatrics, Kennedy Krieger Institute, Johns Hopkins School of Medicine, Baltimore, MD, United States

**Keywords:** hypoxic-ischemic brain injury, inflammatory responses, maternal cigarette smoke exposure, n-acetyl cysteine, sildenafil, Src family kinases, umbilical cord blood mononuclear cell treatment, therapeutic strategies

Perinatal asphyxia and related hypoxic-ischemic encephalopathy (HIE) remain among the leading causes of mortality and significant long-term neurological morbidity with an incidence of 2-4/1000 full-term and 40-148/1000 premature births (Honeycutt et al., 2004; Hilton et al., 2006; Barrett et al., 2007; Fatemi et al., 2009; Scafidi et al., 2009; Higgins et al., 2011; Hagberg et al., 2015). Neonates exposed to hypoxia-ischemia (HI) injury can have poor neurological and behavioral outcomes including cerebral palsy (CP) and incidences of learning deficits and disabilities due to the diffuse nature of the insults. Consequently, developmental disabilities place a huge burden on families, health systems, and society (estimated lifetime costs per person: ~1 million dollars), emphasizing the urgent need for identifying the causes and mechanisms underlying HIE and improved prevention/treatment strategies to reduce perinatal brain damage (Honeycutt et al., 2004). In this Research Topic, we summarized original research and review articles that examine the more recent progress in pharmacological and cell therapies for perinatal HI brain injury.

Risk factors predisposing to HIE can be antenatal, intrapartum, postpartum, or a combination of all. However, the causes of HIE remain unidentified in more than half of the cases (Nelson et al., 2012; Aslam et al., 2019). Maternal cigarette smoke exposure (SE) during pregnancy is a well-documented risk factor that can worsen brain injury and neurological outcomes in adolescent offspring (Reeves and Bernstein, 2008; Chan et al., 2017). Recently, accumulating clinical data suggest robust sex differences in HIE, with male infants showing a higher incidence of injury and more severe long-term cognitive deficits compared to females with matched degrees of injury (Costeloe et al., 2000; Lauterbach et al., 2001; Donders and Hoffman, 2002; Tioseco et al., 2006). The research by Huang et al. investigated the modulation of the effect of SE by sex of the offspring. Interestingly, they observed behavioral deficits and neuronal loss caused by HI brain injury in female adolescent offspring after SE (Huang et al.). In their work, the authors showed that maternal SE worsened the deficits in neurobehaviors such as short-term memory and motor functions, reduced neuronal density and synaptic markers such as ELFN2 and PSD95, and exacerbated glial activation in adolescent females with neonatal HI brain injury, which may be driven by the exaggerated oxidative stress and inflammatory responses similar to their male littermates, as shown in their previous study (Chan et al., 2017). Thus, avoiding maternal SE during pregnancy is important to protect the brain from perinatal brain injuries.

There is now a strong therapeutic basis to treat full-term infants with moderate HIE with hypothermia when started within 6 h of birth, but the degree of neuroprotection remains incomplete, and preterm infants with HIE are excluded from hypothermia therapy. Therefore, there remains a need for additional therapies for the preterm population. (Gunn et al., 1997; Shankaran et al., 2005, 2012; Gluckman et al., 2006; Higgins et al., 2006; Stephens et al., 2010; Perrone et al., 2012; Shankaran, 2012). These therapies should target the cellular mechanisms that underly HI brain injury, including early phase propagators of injury such as neuro-inflammation, cell death, mitochondrial dysfunction, oxidative stress, and excitotoxicity, and they should be safe for use in both term and preterm infants. Xenon or erythropoietin (EPO) are two of the most attractive adjunct therapies. However, treatment with xenon or EPO alone or combined treatment with hypothermia did not improve functional outcomes in infants with severe HIE and extremely premature infants (Ruegger et al., 2018; Oorschot et al., 2020).

The research conducted by Qiu et al. presents a promising therapy that used inhibitors of the Src family kinases (SFKs) for neuroprotection in a preterm model of HI brain injury (Qiu et al.). They induced HI brain injury in postnatal 3-day Sprague-Dawley (SD) rats, which corresponds to 23–32 weeks of human gestation (Mallard and Vexler, 2015). They found that the {4-amino-5-(4-chlorophenyl)-7-(t-butyl) pyrazole [3,4-d] pyrimidine} (PP2), an SFK inhibitor when given within 0.5 h from HI, ameliorated pathological changes and myelin deficits and improved neuro-behaviors in the preterm rats with exposure to HI brain injury. Therefore, targeting SFKs could provide an additional neuroprotective approach to treating preterm infants.

The research by Singh-Mallah et al. focuses on early immune responses which are known to occur within minutes after exposure to HI in the neonatal brain (Algra et al., 2013; Li et al., 2017; Ziemka-Nalecz et al., 2017). HI stimulates the release of damage-associated molecular patterns (DAMPs), which activate pattern recognition receptors (PRRs) to stimulate initial inflammatory responses (Hagberg et al., 2015). Novel therapeutic target molecules such as pro-inflammatory cytokines and DAMPs are continually being identified. The high mobility group box-1 protein (HMGB1) serves as a DAMP molecule and is an early biomarker and pro-inflammatory mediator of brain ischemia (Kim et al., 2006; Liu et al., 2007; Singh et al., 2016). After exposure to HI, HMGB1 is released from brain cells into the extracellular space, where it stimulates and initiates the early stages of sterile inflammation (Kim et al., 2006; Liu et al., 2007; Qiu et al., 2008). Sirtuin-6 (SIRT6), a member of the sirtuin family of NAD (+)-dependent histone deacetylases, has been shown to protect the brain from cerebral ischemia/reperfusion injury *via* regulating inflammatory responses such as the extracellular release of HMGB1 (Zhang et al., 2017). Furthermore, Singh-Mallah et al. used N-acetyl cysteine (NAC), which is an antioxidant and has been previously shown to reduce lipopolysaccharide (LPS)-sensitized HI brain injury (Wang et al., 2007), to further confirm that SIRT6 has protective effects on HIE, which is at least partially associated with the regulation of HMGB1 extracellular release since NAC restores SIRT6 and decreases HMGB1 release in LPS-sensitized neonatal brain following HI injury (Singh-Mallah et al.). Therefore, targeting HMGB1 and its attendant sterile inflammatory responses could represent a novel adjunctive therapeutic approach to treat HIE.

Recently, phosphodiesterase type-5 (PDE5) has gained increasing attention as a potential therapeutic target for several central nervous system diseases, including adult stroke and neonatal HI brain injury (Eun et al., 2000; Chen et al., 2017; Soares et al., 2019). PDE5 expresses in cerebrovascular endothelial cells, neurons, and glial cells (Teich et al., 2016). Sildenafil, a highly potent selective inhibitor of PDE5, is commonly used clinically to treat both term and preterm infants with pulmonary artery hypertension (Samiee-Zafarghandy et al., 2014; Martinho et al., 2020). Interestingly, sildenafil has been demonstrated to cross the blood-brain barrier and has been shown to exert neuroprotective effects based on the evidence of restoring neuronal development, preventing neuronal cell death, reducing neuro-inflammation *via* reducing reactive astrogliosis and macrophage/microglial activation, promoting functional recovery and mediating blood-flow redistribution after neonatal HI (Charriaut-Marlangue et al., 2014; Gomez-Vallejo et al., 2016; Moretti et al., 2016; Yazdani et al., 2016, 2021; Engels et al., 2017). Moreover, maternal treatment with sildenafil is anti-oxidative and prevents neuronal death in an animal model of fetal ischemia (Ozdegirmenci et al., 2011). A comprehensive up-to-date review of sildenafil studies conducted on perinatal HI brain injury were reported in the review article by Xiong and Wintermark.

An exciting new frontier in medicine has been ushered in recent years that applies cell-based therapies to treat neurological diseases. Neural, mesenchymal stem cells and multipotent adult-derived progenitor cells (MAPCs) have been found to improve the repair of the damaged brain (van Velthoven et al., 2009; Bennet et al., 2012). Human cord blood mononuclear cells (hUCBCs) contain a large population of stem and progenitor cells (Penny et al., 2019). The administration of hUCBCs has been demonstrated to reduce neuronal apoptosis, neuro-inflammation, and white matter brain injury in perinatal HI (Aridas et al., 2014; Li et al., 2016; McDonald et al., 2018). Some clinical trial studies showed that hUCBCs therapy improved motor function and cognitive scores in children and adolescents with CP (Min et al., 2013; Kang et al., 2015). The research by Lyu et al. aimed to assess the therapeutic use of hUCBCs and its two components mononuclear cell (MNC) and red cell fraction (RCF) for the treatment of neonatal rat HI brain injury, focused in particular on the analysis of short- (7 days after cell administration) and long- (1 and 3 months after cell administration) term behavioral and neuropathological outcomes. They found both hUCBCs, MNC, and RCF have short- and/or long-term protective effects in neonatal rats with HIE (Lyu et al.). The article by Lyu et al. highlights that treating perinatal HIE with hUCBCs and its components is promising and can be fast-tracked to clinical trials when its availability, dosage, and timing are characterized.

Together, the investigation of additional mechanisms underlying HIE and molecular targets will continue to be essential for the development of new therapeutic strategies that produce more effective treatments for HIE. We believe that the experimental discoveries and opinions presented in this Research Topic will have a major impact on basic and translational research in perinatal brain injury and inspire novel ideas in the future study.

## Author contributions

XC and SM wrote the editorial. JP, LW, and JS revised the editorial. All authors listed have made substantial contributions to the Research Topic and approved it for publication.
